# Smart Assistive Architecture for the Integration of IoT Devices, Robotic Systems, and Multimodal Interfaces in Healthcare Environments

**DOI:** 10.3390/s21062212

**Published:** 2021-03-22

**Authors:** Alberto Brunete, Ernesto Gambao, Miguel Hernando, Raquel Cedazo

**Affiliations:** 1 Centre for Automation and Robotics (CAR UPM-CSIC), Universidad Politécnica de Madrid, 28006 Madrid, Spain; ernesto.gambao@upm.es (E.G.); miguel.hernando@upm.es (M.H.); 2 Department of Electrical, Electronical and Automatic Control Engineering and Applied Physics, Escuela Técnica Superior de Ingeniería y Diseño Industrial, Universidad Politécnica de Madrid, 28012 Madrid, Spain; raquel.cedazo@upm.es

**Keywords:** AAL, smart environments, assistive, robotics, IoT, low mobility, multimodal interaction, eye tracking

## Abstract

This paper presents a new architecture that integrates Internet of Things (IoT) devices, service robots, and users in a smart assistive environment. A new intuitive and multimodal interaction system supporting people with disabilities and bedbound patients is presented. This interaction system allows the user to control service robots and devices inside the room in five different ways: touch control, eye control, gesture control, voice control, and augmented reality control. The interaction system is comprised of an assistive robotic arm holding a tablet PC. The robotic arm can place the tablet PC in front of the user. A demonstration of the developed technology, a prototype of a smart room equipped with home automation devices, and the robotic assistive arm are presented. The results obtained from the use of the various interfaces and technologies are presented in the article. The results include user preference with regard to eye-base control (performing clicks, and using winks or gaze) and the use of mobile phones over augmented reality glasses, among others.

## 1. Introduction

Ambient assisted living (AAL) environments are currently a key focus of interest as an option for assisting and monitoring disabled and elderly people while providing a healthy and independent lifestyle [1] in domestic, professional, and public areas. These environments usually consist of IoT (Internet of Things) networks (increasingly wireless) that connect smart devices, robots (manipulators and mobile), and intelligent interfaces with users. In these smart spaces, a new intuitive and immersive interaction between the environment and its devices, robots, and users is necessary.

Disabilities that result in limitations to mobility can lead to a loss of personal autonomy in a person’s daily life. Increasing the devices and interfaces that patients can control and interact with can help reinforce their independence and therefore their quality of life. In fact, the use of assistive robots can make it significantly easier for a person to carry out their daily tasks. This improvement also reduces the need for family caregivers and medical staff, thereby gaining more efficiency in terms of time dedicated by and costs from health services.

The interaction between a smart environment, assistive robots, and the user gains special importance when it assists people with reduced mobility, limited cognitive capacity, or chronic illnesses that create dependency [2]. To guarantee adequate functioning of robotic care and rehabilitation systems, it is necessary to create a system that can guarantee interactions between the patient, the caregiver, assistive robots, and the IoT environment in a simple and effective way. In the case of patients with special needs, this type of interaction is particularly relevant because it can contribute significantly to improvement in their quality of life. However, it is also necessary to have different ways of controlling the environments, depending on the user’s needs.

The main objective of this paper is to present a new architecture that integrates IoT-based smart assistive environments and robotic systems, together with a new multimodal interaction system: tactile, eye-tracking, gesture, voice, and augmented reality control. The proposed architecture interconnects three worlds that have until now existed separately in the assistive scenario: IoT environments, robots, and humans. It is part of the Robohealth project [3].

The architecture is intended to control the different elements of a room, so the patient can customize their roller-shutters, adjust the bed tilt, and turn the lights on or off. It also gives an opportunity to caregivers to monitor everything remotely (temperature, humidity, door status, lights, and movement). This architecture is comprised of an assistive robotic arm, an IoT network, and a tablet PC with several interfaces: eye-tracking, gesture-tracking, voice recognition, and augmented reality. With this architecture, patients with different disabilities can control the room in different ways: quadriplegics or people who cannot move both arms can use the eye-tracking systems, blind people can use the gesture-based system, deaf people or people with disabilities that affect their speech have the option to use all of the non-voice-based systems, etc. The idea is to have several options, so the user can choose the one that best fits their needs.

The main interface is based on a tablet PC held by an inherently safe assistive robotic arm. When the user makes a command, the robotic arm places the tablet PC in front of the user, so the user can control the room using their hands or the eyes. The user can control the room via voice commands or gestures whenever they want. The architecture is also provided with a ROS (Robot Operating System, https://www.ros.org/, accessed on 21 March 2021) interface to integrate service robots. Mobile robots are increasingly being used in the health ecosystem and can help the user in many ways [4,5,6]. In this architecture, the robot can communicate with the IoT devices in the environment, performing several tasks such as adjusting the light intensity or the bed position.

This paper presents the architecture and the results regarding users´ preferences of the different interfaces. Key aspects of this paper include the IoT–robot interface and, with regards to the multimodal interface, the use of the eye-tracking interface.

The paper is structured as follows: in Section 2, the state-of-the-art is presented. In Section 3, the smart room concept is explained, followed by explanations of the robotic arm (Section 4) and the multimodal interfaces (Section 5). The results are discussed in Section 6, and finally, the conclusions and future work close the paper in Section 7.

## 2. State-of-the-Art

The use of smart assistive environments aims to improve the quality of life of the elderly and people with disabilities. Stojmenski et al. analyzed the possibilities and methods for using assistive technologies, with a focus on the people who cannot regularly control different aspects of their home environments [7]. They proposed a platform that enables end-users of the system to complete everyday activities without additional assistance.

Several architectures that integrate service robots into smart assistive environments have been developed over the last years. First attempts were carried out through PEIS Ecology [8] and the Steward Robot by Park et al. [9]. The ASTROMOBILE project [10] aimed to develop and demonstrate that a smart robotic mobile platform for indoor environments with an embodied bidirectional interface with the world and the users can be conceived to improve services useful for humans. The smart environment was composed mainly of several Zigbee devices and Internet and multimedia applications. Matsumoto et al. integrated humans and assistive robots into an ECHONET-based smart home architecture [11] through a multimodal user interface (focusing mainly on verbal and nonverbal interactions) [2]. Its main goal was to mitigate nursing care problems in aging societies. However, in recent years, the number of platforms combining robots and smart environments has increased, as shown by Anghel et al. [12].

Additionally, robotic arms are being increasingly used for assistance tasks. Leaving aside the purely commercial area (for economic reasons), in which it is possible to find Universal Robots or Kinova robots, there are other interesting projects. Marquez et al. presented a care robot with the capacity to change autonomously between different available manipulators such as a spoon, a fork, and a bowl [13]. In the article, the authors commented that an intuitive system is more easily accepted by the users, which implies that the users will sooner begin to use the facilities offered by a robot of this type. A folding robotic arm that can be stored or transported in a suitcase is presented by Hideyuki et al. [14]. It comes with a handheld manipulator that allows for gripping a wide range of objects.

Along this same line, it is possible to find other models to help with feeding [15], designed specifically for people with reduced mobility [16] or for care purposes [17]. In the study presented by [18], various models of assistive robotic arms were compared in terms of mobility, load capacity, and safety.

The existence of numerous wireless protocols and networks has always posed a problem for interoperability. However, it is an issue that needs to be addressed since there has never been, or is there expected to be, a clear winner in the wireless protocol arena. For this reason, most architectures propose the use of a multi-protocol gateway that allows for the coexistence of various wireless protocols. For example, Echonet offers eight options for its transmission media, including power-line communication (PLC), low-power wireless, IrDA (Infrared Data Association) control, Bluetooth, Ethernet, wireless LAN (Local Area Network), and 802.15.4 with 6LoWPAN [11]. A suitable medium depends on the environment of the user, so the option chosen will have weak points depending on the structure of the house or radio environment. DOMHO [19] entails multiple network technologies (Bluetooth, Wi-Fi, ZigBee, and LoRaWAN) through the DOMHO gateway, and a variety of user control interfaces. For the purpose of this paper (control smart-home devices in a room), most wireless networks (Wi-Fi, Zigbee, Z-Wave, and Bluetooth) comply with the technical requirements in terms of latency, payload, and data rate [19]. Other authors [20] even conclude that 5G networks will be effective in similar healthcare scenarios, such as telesurgery, remote pervasive monitoring, and m-health.

The importance of multimodal interaction was described by Sharma et al. [21] and has been since then increasingly used in assistive environments. It is a way to make user interfaces natural and efficient with parallel and synergistic use of two or more input or output modalities [22]. Since its beginning, it has been a very important topic, but up until the last decade, the most widely used interfaces for human–computer communication were peripheral devices, such as a keyboard or a mouse. [23]. However, these kinds of devices are currently insufficient to control all devices in a smart room. Speech, gestures, face, and many other modalities appear to be more efficient and natural ways to interact with our environment. For example, multimodal interfaces have been used with robots, from buttons and voice [24] to eye tracking [25], even decoding the gaze position in 3D space [26].

However, it is the use of smartphones and tablets that has provided a neat interface with which to control all devices inside a room or house. Now, with the arrival of Google Home and Amazon Alexa [27,28], voice control is gaining a lot of popularity. Making use of these services, Nasr et al. describes a scalable and expandable system with the goal of designing a natural speech-enabled system for older adults [29].

Although important, tactile and voice interfaces are not always suitable for all people. People with reduced mobility due to injuries or accidents need different ways in which to interact with the environment. A multimodal interface may offer different solutions to people with different needs. Combining tactile, eye-based, gesture-based, and voice control allows all users to control their automated rooms seamlessly.

Different research works are studying how to integrate new ways of interaction with mobile devices such as smartphones and tablets to provide a natural and easy mode of communication [30]. Touch and voice control is the default way, but new ways are appearing, for example, eye tracking, which is probably the newest interface method. It has been used in [30] to monitor tablets through head motions and eye-gaze gesture recognition. This system can open a browser application with simple motions of the head and the eyes.

Eye movements are preserved in many movement disorders leading to paralysis (or amputees), including stroke, spinal cord injury, Parkinson’s disease, multiple sclerosis, and muscular dystrophy [25]. Maimon et al. demonstrated how combining 3D gaze tracking with a custom-designed 3D head tracking system and calibration method enables continuous 3D end-point control of a robotic arm support system [25].

Winks can be an interaction method with the environment. It is possible to detect eye winks with up to 98% accuracy [31] (using special glasses). Noronha at al. explored the use of binocular eye-tracking technology to detect voluntary winks from involuntary blink commands and to establish winks as a novel low-latency control signal to trigger robotic action [32]. They also compared their approach to two conventional approaches for controlling robotic devices based on electromyography (EMG) and speech-based human–computer interaction technology, concluding that their eye-tracking-based approach to control assistive technologies is a well-suited alternative to conventional approaches, especially when combined with 3D eye-tracking-based robotic end-point control.

Gesture-based control has been used not only for arm robot control [33,34], mobile robots [35], and Unmanned Aerial Vehicles (UAVs) [36] but also as an interface to control smart rooms. Gesture-based control can be divided into visual-based recognition, 3D-camera-based recognition (including Microsoft Kinect), and sensor-based recognition (including wearables). Vision-based gestures include mainly hand gestures. Lamb et al. [37] controlled the position of a bed automatically by using hand gesture recognition based on image processing algorithms [37], and Ransalu et al. used it to control smart appliances [38]. Regarding 3D-camera-based recognition, Handosa et al. used the Kinect sensor to control lights [39]. In sensor-based recognition, Czuszynski et al. [40] used a contactless linear gesture sensor for the navigation of a smart lighting system to propose two methods of active light source selection: continuous dimming, and turning on and off based on discrete gestures. They achieve a gesture recognition accuracy of up to 97.58%. Petersen et al. [41] described a study showing that 80% of the participants preferred to use gestures over more traditional methods such as GUIs. The participants had no problem completing their tasks after the first or second try.

That being said, two conclusions can be drawn from the state-of-the-art. The first is that there are numerous architectures that are committed to the integration of IoT systems and robots in healthcare environments. There is also a need for an interface that allows their interconnection through different protocols, given that currently there is not an interface that dominates the market. Second, all the user interfaces considered (eyes, voice, gestures, augmented reality (AR), and touch) are important and have a great amount of literature behind them. Their coordinated use can help people with different disabilities. Consequently, this paper aims to contribute on both sides: a multi-protocol architecture that allows the integration of devices of different technologies and robots in a transparent way, and a multimodal interface that allows access to people with different disabilities.

## 3. System Architecture

**Figure 1:**
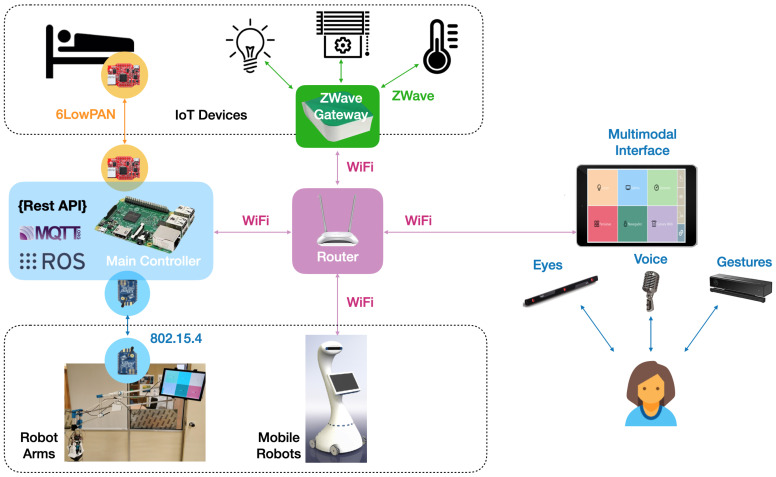
Network architecture.

The system architecture presented in this section is designed to allow for the interconnection and cooperation of different IoT networks, multimodal interfaces, and service robots. Figure 1 illustrates a block description of the network infrastructure, and it is explained in the following paragraphs.

Because many IoT protocols cohabitate today, the architecture must be able to interconnect different protocols. At this moment, the following technologies have been selected: Wi-Fi communications, Zwave communications (via the Z-Wave Vera gateway), 6LoWPAN (IPv6 over Low-Power Wireless Personal Area Networks) communications (via a Zolertia Z1 border router connected to the Raspberry Pi), and 802.15.4 communications (via a XBee module).

Additionally, in order to interconnect these devices with service robots, a common interface was designed.

### 3.1. Main Controller

Although the architecture presented is distributed and each device can operate independently, in order to create a common interface for all devices, a main controller was designed. This main controller gathers information about all the devices connected in the smart room and provides an open interface to visualize and control them, regardless of the technology they use.

**Figure 2:**
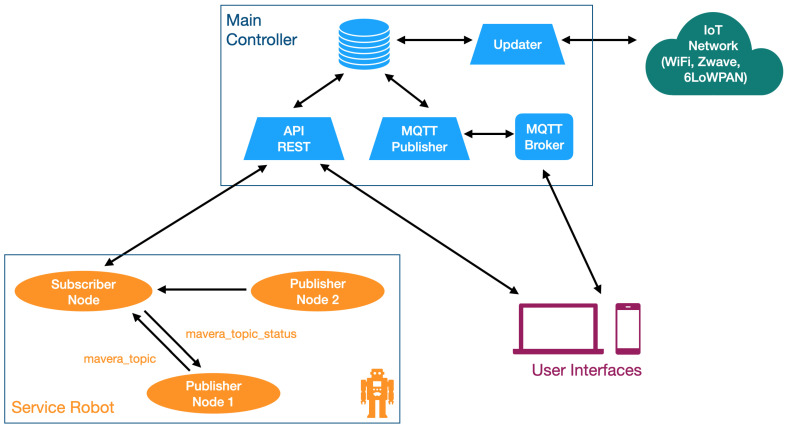
Block diagram of the main controller.

A Raspberry Pi (model 3B/4) was chosen to act as the main controller due to its good quality/price ratio. It runs a Node-RED application that is in charge of three main tasks (Figure 2).

The first one is to run the updater, a process to monitor the state of all devices, updating a database that can be accessed by other processes. In particular, it performs the following actions:

- control the physical devices connected to the Zwave network as well as the visualization of their data via the VeraLite controller, which controls the Zwave network. The connection with the VeraLite controller is by TCP/IP (Transmission Control Protocol/Internet Protocol) and REST (REpresentational State Transfer) commands;
- control the physical devices connected to the 6LoWPAN network via a border router connected via USB, which allows communication with the 6LoWPAN wireless sensor network;
- control devices connected to the 802.15.4 networks (like the robotic arm) thanks to the Xbee module, connected via USB; and
- control the physical devices connected to the Wi-Fi/Bluetooth network;

The second task is to provide an MQTT (Message Queuing Telemetry Transport) interface to allow service robots and multimodal interfaces to control the IoT networks. MQTT is an open protocol that is extensively used in the world of IoT. The Raspberry Pi runs an MQTT Broker and publishes new messages whenever the value of a device changes (i.e., temperature, light, or shutter). Service robots and multimodal interfaces can subscribe to these topics in order to obtain these values.

The third task is to provide a RESTful interface to allow service robots and multimodal interfaces to control the IoT networks. RESTful services are also extensively used in the Internet world and are available on most devices. Thanks to this interface, service robots and multimodal interfaces can obtain information about the network through HTTP (i.e., GET and POST) commands.

### 3.2. IoT Interface

The Internet of Things (IoT) infrastructure is composed of different sensor and actuator networks interconnected by a common gateway/controller. Currently, the following standards can coexist simultaneously: Z-Wave, 6LowPAN (Contiki), Xbee-based (802.15.4), Wi-Fi, and Bluetooth. However, any other protocol can be added in the future (as long as it is open or has an open API). This IoT network can support sensors suhc as humidity, light intensity, temperature, movement, and reed (for the main door). Actuators include automated lights (on/off and regulation) and roller shutters.

The backbone network is a Wi-Fi network. All networks are interconnected via Wi-Fi. In the case of IoT networks different from Wi-Fi, they need a gateway to connect to the Wi-Fi network. The following networks are available: ZWave, 802.15.4, and 6LowPAN. Both commercial (ZWave) and research (802.15.4 and 6LowPAN) networks were considered.

The Zwave network connects Zwave devices: lights; shutters; and water, temperature, and door sensors. It is based on commercial devices.

The 6LowPAN network is based on Zolertia Z1 motes running the Contiki OS. They are used to control the bed and to monitor its position. The bed is an electrically adjustable model that has been modified to connect it to the network. The head and feet parts were sensorized and the motor can be remotely actuated by a relay, but the original wired remote controller of the bed remains operative at any moment. The bed system becomes consequently another node of the room. These modules form a small sensor network, with one of the modules (connected to the Raspberry) acting as the border router.

The Xbee-based network is used to communicate with the robotic arm shown in Section 4. It uses the 802.15.4 protocol (as well as 6LoWPAN in its lower levels), which is a light low-consuming protocol.

Finally, the Wi-Fi network is used to communicate the main controller (Raspberry Pi) with the Zwave controller (VeraLite), the tablet PC, and other devices or robots in the room.

The use of IoT devices enables monitoring from anywhere in the world: this allows caregivers or family members to monitor the patient’s use of the room and to provide assistance remotely in case of technical problems.

### 3.3. Robot Interface

Service robots can connect to the architectures as long as they have a Wi-Fi interface. We built a ROS interface for that purpose. Publisher and subscriber nodes were implemented in ROS. The subscriber node is in charge of communication with the main controller via REST commands to get information or to modify the values of the actuators. Other publisher nodes running on the robots can publish messages that will be received by the subscriber node. Consequently, publisher nodes can interact with the IoT network seamlessly. Two topics are used: mavera_topic is used to publish commands (publisher to subscriber) and mavera_topic_status is used to publish the status of the sensors (subscriber to publishers). Figure 2 shows the node-topic structure.

### 3.4. User Interface

A multimodal interface was designed to allow the user to interact with the environment by different means. It is composed of a tablet PC equipped with an eye-tracker and a Microsoft Kinect camera. The tablet PC provides tactile control, voice control, eye-based control (thanks to the Tobii Eyex device connected to the tablet PC) and gesture control (thanks to the Kinnect device connected to the tablet PC).

One of the most relevant features of this architecture is the ability to control the room and the robots in a multimodal way. For this purpose, the interaction is being mainly developed for a high-performance tablet (Surface 4), with access to the IoT network and the robotic arm. With this tablet PC, it is possible to control them by means of the following:

- touch: provided directly by the tablet. The GUI will emit events as a result of the interaction with its elements on the touch screen;
- eye control: based on the data given by the device Tobii EyeX, the system is able to react to commands made by the user’s gaze and winks;
- voice control: based on the Sphinx library, it allows for control of the room with voice commands (equivalent to the commands written in the tablet PC); and
- gesture control: based on the 3D Kinect sensor by which the system is able to react to gestures made by the user.

**Figure 3:**
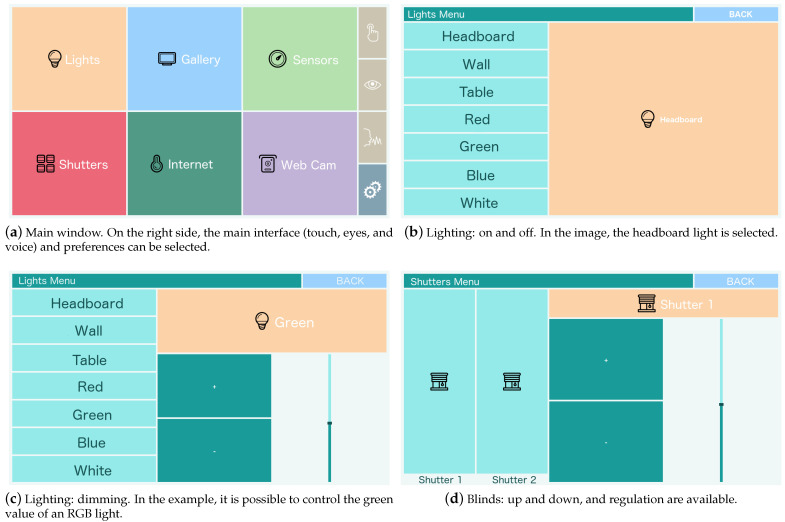
Different views of the tablet interface.

Figure 3 illustrates the application that runs on the tablet PC. In the main screen (Figure 3a), it is possible to access up to six menus: light control, shutter control, sensors display, photo gallery, browser, and webcam, but they can be configured. It is also possible to see the different control modes, starting from the top: tactile, eye-based, and voice-based. The last button is the configuration, gesture-based control, is always activated. Figure 3b,c show the light control and Figure 3 the blind control.

Apart from these interfaces, an augmented reality (AR) interface for the mobile phone or AR glasses was also developed. This one, with eye-, gesture- and voice-based controls, will be explained in Section 5.

## 4. Assistive Robotic Arm

Most commercial robotic arm models are expensive and have features that exceed those needed for this application in which the user simply needs to position a tablet PC in front of himself or herself.

The arm presented in this article is designed to support as its end-effector a tablet that could be positioned in several predefined positions, allowing the patient to use easy interactions through voice, gestures, eye-tracking, or touch screen commands. The tablet should always be at a specific distance and angle from the patient’s face (for eye-tracking).

The main objective is to guarantee the patient’s safety so that the arm cannot exert any force that might harm the patient. With this in mind, an actuator system based on gravity-balanced wires and springs-based links has been designed. The patient can move the arm in any direction by pushing it. It is designed mainly for patients with reduced or no mobility (temporary or permanent), although it could also facilitate the human–room interaction for any user.

### 4.1. Design Requirements

**Figure 4:**
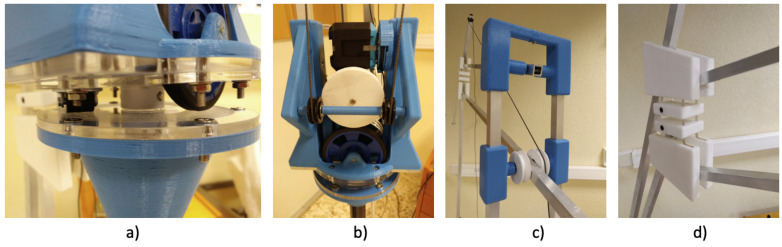
Robotic arm joints in detail: (**a**) joint 1, (**b**) motors and pulleys, (**c**) joint 2, and (**d**) joint 3.

The arm has been designed with the following principles in mind:

- The arm should be low-cost.
- The arm must be able to support, in all its ranges of movement, the weight of the tablet PC (Microsoft Surface model), with a weight of around 770 g.
- The use of motors with low torque is required. In this way, it becomes physically impossible for them to pose a risk to users since the force they are able to generate is not sufficient to pose a danger.
- The use of low-torque motors implies a compensation of the weight of the arm itself so that the motors must be loaded with the lowest possible weight. This is achieved through mechanisms of four coupled bars.
- The choice of these motors implies a mechanical amplification in the transmission of the movement. The motors are located in the base, and the torque is transmitted to the joints by threads. This lowers the inertia of the arm and eases gravitational compensation. The transmission works only in one direction, that is, to raise the end of the arm. The opposite direction will be achieved by releasing the thread and by letting the weight of the arm lower it down. The patient cannot suffer any damage if he or she is in the trajectory of the robot because it is not able to exert an active force on the patient. The only load supported will be the weight of the tablet once the load is compensated.
- As shown in Figure 4a, horizontal movement is made by friction. In this way, in the case of impact, the actuator would slide, ensuring its intrinsic safety.

### 4.2. Electromechanical Design

The electromechanical design includes an Arduino MEGA board, a shield to control the motors (Shield-G15 REV2.0 from Cytron), three motors (G15 Smart servomotors from Cytron), three potentiometers TW1502KA from TE Connectivity (for position control), and a power supply S-120-12. The communication between the Arduino and the motor shield is serial (UART). The communication with the room controller is made by an Xbee module (802.15.4). A set of predefined commands have been created to communicate between the arm and the controller.

The use of G15 Smart servomotors capable of performing up to 20 W with low operating torque makes it physically impossible for them to pose a risk for the users. Additionally, this choice of servomotors implies a mechanic amplification throughout the movement drive in the designed prototype. The motors are placed at the base, and their torque will be transmitted to the joints through wires, the smoothest and most silent option.

### 4.3. Final Prototype

**Figure 5:**
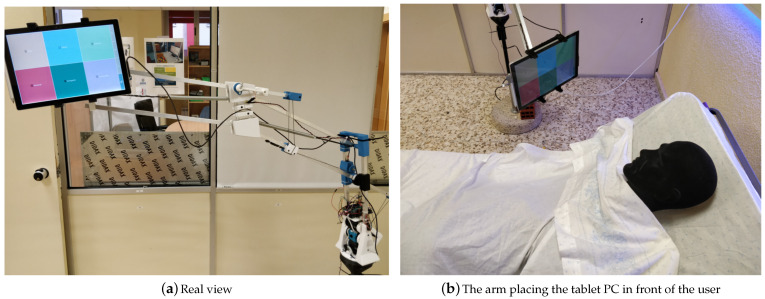
Assistive robotic arm.

Figure 5 illustrates the results of the last version, which has a range of movement of 50 cm on the X-axis and 90 cm on the Z-axis. It is able to carry a load of 780 g. Two repeatability tests were performed; Table 1 shows the results. Although the accuracy is not very high, it is sufficient for application. The cost of the robot arm is around 300 €.

A specific user application was designed for the tablet PC (placed at the end-effector of the robotic arm) to control the room.

## 5. Multimodal Interface

A specific user application was designed for the tablet PC (placed at the end-effector of the robotic arm) to control the room. The four control modes are described below.

### 5.1. Eye-Based Control

In this section, the eye-based control will be described.

**Figure 6:**
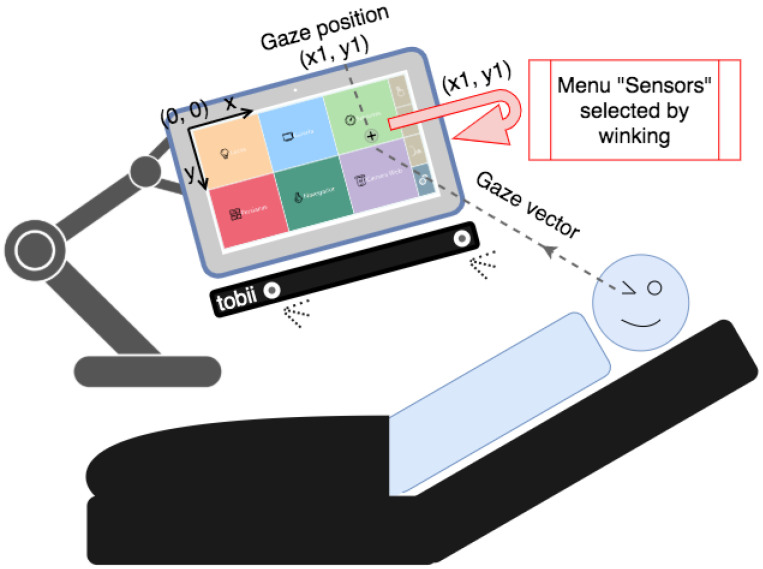
Eye-tracking coordinate system and wink detection.

Eye-based control is based on the Tobii EyeX Controller, an eye-tracking device that uses near-infrared light to track a user’s eye movements and gaze point. This device automatically collects the size of the screen to calculate the point where the user looks. The coordinates where the user is looking are referenced with respect to the upper left corner of the screen, such that the positive X-axis is horizontal to the right and the positive Y-axis is vertical downwards (as shown in Figure 6). To read these coordinates, Tobii EyeX collects the data in a normalized way and converts them into the appropriate pixels where the person is looking. Although this device is intended for video games, we used it as an interface for patients.

In the eye-based control mode, the user can control the room with the same visual interface, but selects a button by looking at it and clicking on the button in two ways: either staring for a specific amount of time or winking. The length of time to be considered a click can be modified by the user from the configuration screen. In addition, in that configuration screen, it is possible to configure the user profile, such as recalibrating the device or changing the user parameters, if necessary.

**Figure 7:**
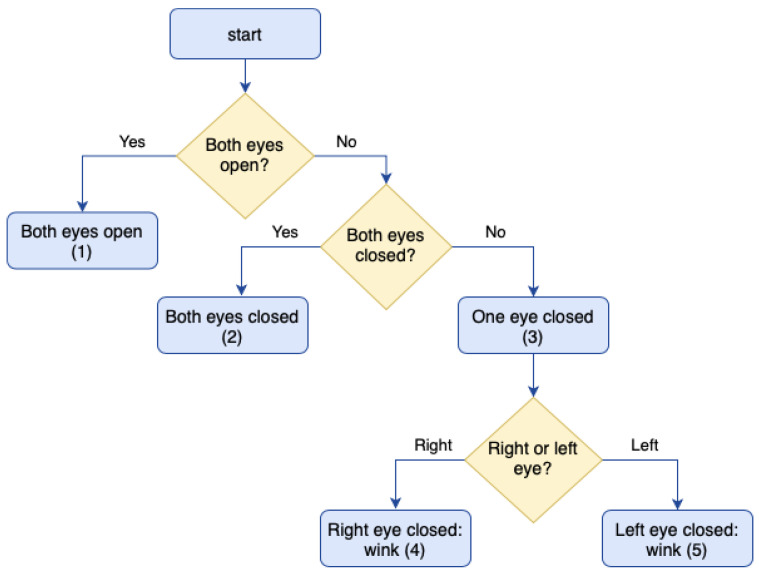
Eye closure detection flow.

The Tobii EyeX can calculate where the user is looking and what is the position of the user’s eyes with respect to the center of the computer screen. Thanks to this, it is possible to compute when the user winks. The Eyex device provides the x and y coordinates of the pixels where the user is looking. However, to click (a button or object), it is necessary to implement this functionality because it is not provided by default. It is an original feature presented in this paper. This is achieved by detecting when an eye is closed for a specific amount of time. The current state of the user’s eyes is monitored continuously. When an eye is closed or the device cannot detect it, the coordinates of that eye will be equal to 0. To do so, the procedure shown in Figure 7 is followed. To determine the time that a wink lasts, the user’s blinking is monitored while the eye mode is not active. When the eye mode is active and one of the eyes is closed for more than the average blinking time (plus an offset), a wink is detected. This is because some people may blink one eye at a time instead of both, as the study in reference [42] concludes.

In Figure 7, state 1 indicates that both eyes are open, state 2 indicates that the user has both eyes closed, state 3 indicates that the user has one eye closed, and states 4 and 5 indicate that the user has winked (right or left eyes, respectively). To be able to consider that, in state 3, there has been an eye closure or that, in states 4 or 5, there has been a wink, it must be verified that they have been kept closed for a specific amount of time. This is done with timers that are triggered when the user has both eyes open and checked when the user has one or both eyes closed. When the user is in any of states 3, 4, or 5, the system will check that the time elapsed between having the eyes open and closed has been equal to or greater than the one indicated in the configuration screen.

The application is prepared to distinguish between right and left winks to perform different actions with each of them. However, in the end, this was not implemented since not all users know how to wink both eyes.

Finally, the user can change the click mode (between staring and winking) by keeping their eyes closed for a certain time. This is done in state 3 when the user keeps their eyes closed for a specific amount of time (more than 2 s). This changes the operating mode and shows it on the screen by means of a label under the “eyes” mode button.

To avoid flickering in the OS pointer, measurements from the Eyex device are passed through a moving-average filter (30 samples) because it was observed that, although the user was staring at a fixed point, the vibrations of the eyes caused a lot of noise in the received data. This caused the screen pointer to vibrate in an excessive way that is very annoying for the user.

### 5.2. Gesture-Based Control

This section describes implementation of the gesture recognition interface for upper limbs. This interface is designed to control the room from the bed. The considered signs have to be made while the user is seated or lying down, and consequently, they should be simple movements.

The key point in the implementation is the efficiency of recognition rather than the variety of signs. A system with only a few signs that are concatenated to achieve a simple task is more adaptable to this use than a system with one sign for each task. Of course, the system’s rate and speed of recognition of the signs must be high enough for the patient to easily send the commands.

The sensor used to detect upper limb gestures is the Kinect v2, developed by Microsoft. The SDK (Software Development Kit) used is the official Kinect for Windows SDK. Thanks to the SDK, it is possible to detect signs. A sign is a position of a body, or a part of it, maintained for a given time. Signs are made with the forearms and hands only. In this way, the user can be lying in the bed and can still control the room. The current implementation uses a simple geometrical analysis.

The following gestures have been defined.

1. Turn on and off the lighting of the room.
2. Open and close the blinds.
3. Request attention or move the robot in front of the user.
4. Raise and lower the backrest of the bed.

**Figure 8:**
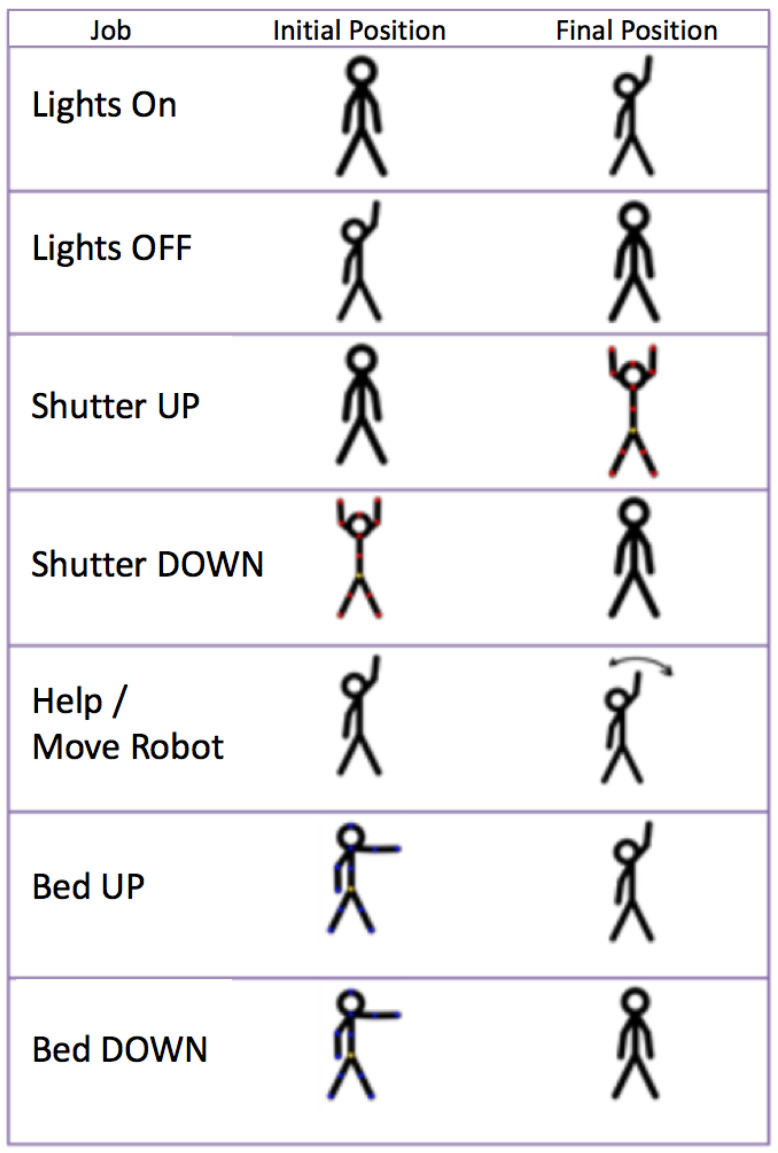
Gestures.

Figure 8 shows the gestures. The gestures were selected after polling several users to identify the gestures that best matched the function they were mimicking from the user’s point of view.

All gestures start with a common sign: right arm fully stretched to the Kinect with an open hand. For analysis of this type of gestures, an algorithm has been developed that detects the starting position of the gesture and, after an estimated time suitable for the transition of an average gesture, picks up the final position. In this way, through the combination of initial and final positions, we obtain valid results for adoption of the determined actions.

A special sign is used to communicate with the robotic arm: right arm fully stretched to the Kinect with a closed hand. Using this sign causes the robotic arm to move the computer in front of the patient.

### 5.3. Voice-Based Control

This control mode works using voice commands. These pre-programmed commands match the text that appears on each screen. For the program to start listening to the user, it is enough to say a keyword. In this case, the word “Larry” has been defined (not a common word in Spanish), and then, the user must say the desired command aloud. This will lead to a virtual click on the button corresponding to the spoken command, therefore performing the function that the button implements.

The Pocketsphinx library (https://cmusphinx.github.io/, accessed on 21 March 2021) was used to develop the voice recognizer. For operation of the Pocketsphinx library, different files are necessary for the recognizer to work. There is an acoustic model and a dictionary of the language in which the recognizer will work. In addition, for this project, the decision was made to limit the recognition to a series of predetermined commands within several “grammar” files because the intention of the project is to implement a controller for which the user will only use a few words and not a dictation application in which the user could say any word. That is why the words susceptible to being recognized were bounded, thus increasing the reliability of the recognizer.

The program begins to listen from the moment it detects the key word until enough time passes to be considered silence, that is, that the person has finished speaking. This waiting time is a pre-programmed value within the library and is invariable. Once this end of “speech” is detected, the received audio is compared with the configuration database, and the hypothesis recognized in a variable is stored.

### 5.4. Augmented Reality Interface

Finally, an augmented reality interface was developed for the system. It does not run on the tablet PC but on Android devices.

**Figure 9:**
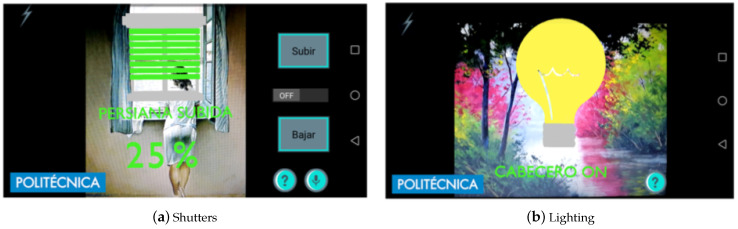
Augmented reality interface.

It is composed of an Android application running on mobile phones or augmented reality glasses (such as the Moverio glasses) and uses some paintings placed all around the room as tags where the information is displayed. When the user looks at the paintings wearing the AR glasses or points at them with the mobile phone, the user can see images overlapping the paintings, as shown in Figure 9. The user can interact with these images, for example, switching the light on and off or moving the shutters up and down.

The application was developed on Android using the Vuforia library for AR. Paintings by well-known artists were selected as image tags. Besides serving as an element to control the room, they also decorate the room.

## 6. Results and Discussion

The architecture was tested on a platform in a laboratory environment, and the obtained results are presented in this section. First, the demonstrative room is presented. Second, the participants that have tested the platform are described. Finally, the results of the tests are presented and discussed.

### 6.1. Smart Room

A demonstrative room that allows the integration of robots, sensors, actuators, and interfaces of different technologies was built. This room allows users with reduced mobility or speaking limitations to interact with the devices in the room in different ways.

**Figure 10:**
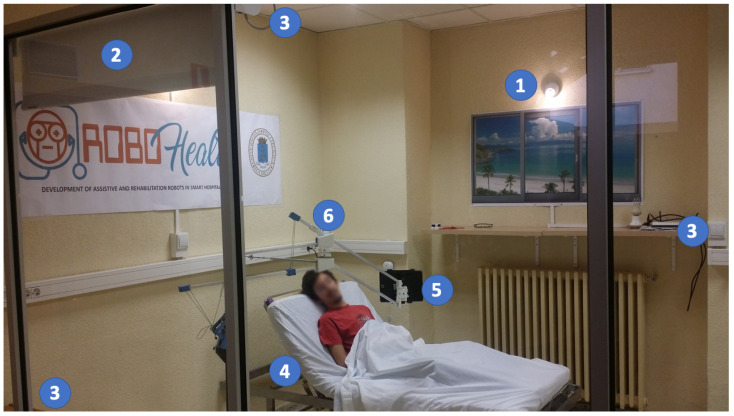
Room.

This smart room allows the patient to experiment with the different devices they can control and interact with. Figure 10 illustrates the test room. The smart room offers the possibility to control lights (1) and shutters (2). The room is equipped with network-connected sensors to monitor the room (3). The bed is adjustable (4), allowing for head and feet elevation. Thanks to a multimodal interface (5), the patient is able to control the room. For those patients with reduced mobility, the system employs a robotic arm (6) that allows the placement of the multimodal interface in predefined positions to facilitate the interaction with the patient (mainly over the bed in front of the user).

Currently, the following networks are operative and working together:

- The Zwave network connects Zwave devices: lights; shutters; and water, temperature, and door sensors. It is based on commercial devices.
- The 6LowPAN network is based on Zolertia Z1 motes running the Contiki OS. They are used to control the bed and to monitor its position. The bed is an electrically adjustable model that has been modified to connect it to the network. The head and feet parts have been sensorized and the motor can be remotely actuated by a relay, but the original wired remote controller of the bed remains operative at any moment. The bed system becomes consequently another node of the room. These modules form a small sensor network, with one of the modules (connected to the Raspberry) acting as the border router.
- The Xbee-based network is used to communicate with the robotic arm. It uses the 802.15.4 protocol (as well as 6LoWPAN in its lower levels), which is a light low-consuming protocol.
- Finally, the Wi-Fi network is used to communicate the main controller (Raspberry Pi) with the Zwave controller (VeraLite), the tablet PC, and other devices or robots in the room.

The platform is operative, and all devices are accessible via the previous networks.

### 6.2. Validation Tests

#### 6.2.1. Participants

The aim of the validation tests was to prove that the application works correctly and that it can be used with patients with a disease in its later stages. Therefore, all tests were performed with students from the Universidad Politécnica de Madrid and their relatives. The tests took place at different stages of the development of the application, so each test was performed with different subjects.

Participants were recruited and screened for admission into the study from the students of the Universidad Politécnica de Madrid and their relatives. Prior knowledge of smartphones, networks, or computer software was not required.

##### Eligibility Criteria

Subjects were included into the study according to the following criteria: (1) age between 20 and 60 years old; (2) no pathologies detected in vision, hearing, and joint movement; (3) not taking medication at that time; (4) basic knowledge (entry level) of informatics regarding the use of a mouse and a keyboard; and (5) informed consent to participate.

#### 6.2.2. Eye-Control Test: Blinking Time

The first experiment was performed in order to determine the length of time needed to consider an eyelid movement as a wink instead of an involuntary blink (and consequently to activate a click).

Ten users were selected, aged from 23 to 25 years old, and 5 women and 5 men. Five of them wore glasses, and five did not.

The user was asked to stare at a black screen with a yellow cross in the center. The application collected the times of the first ten blinks, as shown in Table 2 (for users with glasses) and Table 3 (for users without glasses).

One-way ANOVA did not show any effect or interaction of the use of glasses in the blinking time (*p* > 0.05).

The average blinking time was 0.182 s (0.181 s for users with glasses and 0.182 s for users without glasses).

The confidence interval is [0.152, 0.211]. Anything above 0.22 s is considered appropriate to distinguish between a wink and a blink. However, in order ensure that it is possible to choose the highest value obtained from any user, 0.6 s from user 3 was considered.

#### 6.2.3. Eye-Control Test: User Experience

To evaluate the user’s experience in the eye mode, a test was conducted with fifteen users of ages from 23 to 59 years old, 6 women and 9 men. Four of them wore glasses, 2 wore contact lenses, and 9 used neither. No one was taking medication at that time.

Users were given the following instructions before starting the experiment.

*This application has 4 operating modes (touch, eyes, voice and gestures). In this test, you will only use the tactile and eye modes. The eye mode has two methods of interaction (winking and staring) that can be selected from the screen or by keeping both eyes closed for the specified time (in the configuration window). Please follow these steps.*

*1.*  *When you start the program, click on the connect button to interact with the room.*
*2.*  *From the main screen, press the configuration button and create your own user using the Create User button.*
*3.*  *Add a favorite on the main screen of the application.*
*4.*  *Press the Back button to return to the main screen and, once inside it, press the Eyes button. From this moment, the application can be used only with the eyes. To accomplish this, you must stare at the button you want to press.*
*5.*  *Press the Alert button in the Favourite box of the main screen.*
*6.*  *Access the Actuators screen to observe the changes made.*
*7.*  *Interact with the different actuators until they are all turned off.*
*8.*  *Return to the main screen using the Back button.*
*9.*  *Change the operating mode to Wink mode (to do this, close your eyes for the amount of time you have set in the configuration screen and make sure that the mode has been changed to wink in the main screen) and enter the sensor screen.*
*10.*  *Once all the sensors are observed, return to the main screen and interact with the buttons previously added to the favorite group.*
*11.*  *Activate the Staring mode again (performing the same operation as in the previous step) and, finally, select the Touch operation mode.*

Subsequently, users were asked to complete a survey that asked different questions about their experience. The survey consisted of the following questions, with the answers from the first and the second questions being yes and no and the rest ranking from 1 (hardest, none, and worst) to 5 (easiest, very, and best).

*1.*  *Are the instructions for the use of the application clear and adequate?*
*2.*  *Is the use of the various buttons intuitive?*
*3.*  *How difficult was the interaction with the buttons in the winking-mode?*
*4.*  *How difficult was the interaction with the buttons in the staring-mode?*
*5.*  *How difficult was it to make the change between both modes of operation?*
*6.*  *How well do you think you understand what state each actuator is in?*
*7.*  *How difficult was the interaction with the available predefined users?*
*8.*  *To what degree do you think the application will be useful for patients?*
*9.*  *What overall score would you give to the application after your user experience?*

**Figure 11:**
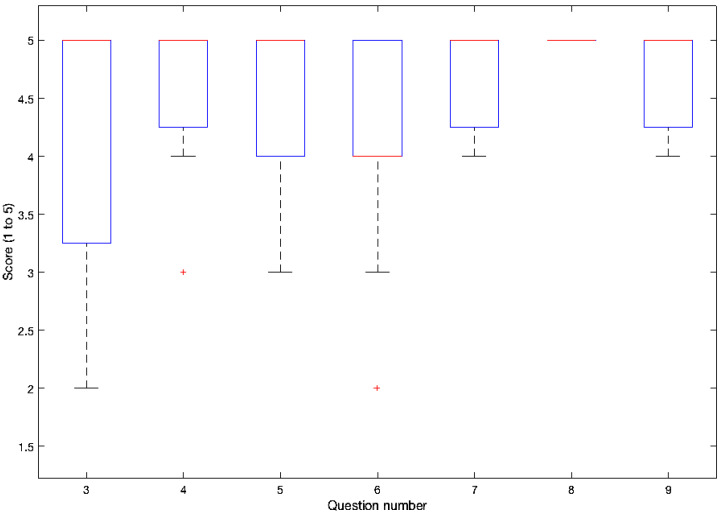
Answers for questions three to nine: one means hardest, none, and worst while 5 means easiest, very, and best.

The answers for questions three to nine are shown in Figure 11. The results of questions one and two (yes or no) were 100% of the users considered the use of the buttons intuitive and 93% considered the instructions clear and adequate.

Although more than 50% of the users found it very easy to interact with the buttons using winking, several users claim that the time needed to consider a wink as a click is too high. Therefore, we consider that the winking time (the parameter that differentiates a wink from a blink) should be configurable by the user in a pre-calibration phase.

Unlike the winking mode, over 85% of the users considered that, in the staring mode, it is very easy to click on the desired button. Consequently, we can conclude that most users prefer the staring mode.

Changing the mode (between staring and winking) by closing the eyes was fairly easy for more than 75% of the users.

Regarding the interaction with the users available in the eye tracker, more than 70% of the users found it very easy to create their own user, while the rest found it quite easy and no user made any complaints about it.

#### 6.2.4. Voice Control Test

To test the performance of the voice control system, four tests were carried out: to turn on the headboard light, to increase an adjustable light by 40%, to visualize the state of the sensors, and to move a blind up by 40%. As an example, for the first action (turning on the headboard light), the sequence between the voice assistant (Larry) and the user is as follows:

User: Larry.

Larry: Hello! What do you want to do?

User: Lights.

Larry: Entering the menu lights. Accept or cancel.

User: Accept

User: Larry

Larry: Hello! What do you want to do?

User: Headboard

Larry: Do you want to turn on the headboard light? Accept or cancel.

User: Accept.

Similar scripts were provided for the other tests: to increase an adjustable light by 40%, to visualize the state of the sensors, and to move a blind up by 40%.

The test was performed on ten users, five women and five men, three times for each person. Ages ranged from 23 to 56 years. These tests were carried out in a quiet environment and at all times using the existing commands on each screen, trying to simulate correct use of the application. In each test, the user interacted with the application following a script. One researcher took notes of the results.

The results of the voice mode showed that, on average, a person with speaking capacity takes approximately 28 seconds to turn on a light, 41 seconds to increase an adjustable light by 40%, 13 seconds to visualize the state of the sensors, and 40 seconds to move a blind up by 40%. Time was measured from the moment the voice mode iwa enabled until the change was observed in the room. The voice recognition library responds better to bass voices than to treble voices. A difference of 3 seconds was obtained between the results of the male and female voices. It is important to note that it is important not to intone the words and to say them with a neutral tone for a better performance. In addition, it has been observed during the tests that a person modulating the voice towards low tones obtains better recognition of the application.

Several issues about the application are interesting. First, when the user uses a command that is not in the list of available commands, it is more than likely that the recognizer confuses it with one of the existing commands. This is due to the nature of the recognizer, since it always searches for a match and therefore takes as a recognized command the one that most closely matches the recorded command. The use of grammar files has an important influence because they limit the commands to a small number. At first, we thought we would not include grammar files and simply looked for matches within the whole dictionary (of more than 3000 words), but it was found that the effectiveness of the recognizer was very low. Therefore, it was decided to use the pre-programmed commands (grammar files), since they considerably improve the quality of the recognizer. The drawback is that they limit the freedom of the user, but in this case, it is not a problem because the set of commands is limited.

Second, it is interesting that the recognizer works in the same way when it is used with different voice tones, since the user can speak lower (provided the user speaks loudly enough to be heard) or higher, and the quality of recognition remains unchanged.

Third, when the application was used in noisy environments, such as when music was playing, people were talking, or devices were making noise in the background, the effectiveness of the recognizer decreases considerably. The most annoying noises for the recognizer are undoubtedly other conversations while it is being used since it usually listens to words and becomes confused very easily, which makes the application unusable through the commands of voice. However, this is not a problem because the user can use the voice commands when he or she is alone. If they are with more people, they can receive help in the tasks they wish to carry out.

#### 6.2.5. Gesture Control Test

Gesture-based controls are used mainly to command the robot to position the tablet in front of the patient and for simple commands such as switching the light on and off.

To evaluate the user’s experience in the gesture control mode, a test was conducted with fifteen users aged from 40 to 60 years old, 7 women and 8 men. No one was taking medication at that time. Users were given the following instructions before starting the experiment: *"When instructed, you should perform the gesture ten times in succession, with a pause between each gesture of one or two seconds"*.

The results show a recall rate of 86% for the light gestures, 50% for the shutter gestures, 80% for the bed gestures, and 62% for the robot gesture.

The test shows good results for simple gestures. Complex gestures or a combination of gestures can confuse people. For example, the gestures to switch the light on and off work well, while the gestures to use the shutters do not work so well. This is because the gesture requires combining the movement of both arms. If both arms are not lifted or lowered at the same time, some recognition error may appear. Generally, the more the users use the system, the better results they obtain. After some training, the results improve in all cases above 90%.

#### 6.2.6. Augmented Reality Test: Recognition Distance

The AR mobile app was tested on different devices: Epson Moverio BT-200 glasses and mobile phones Huawei Honor 8, Samsung Galaxy s7, and Huawei P9.

One important issue in this type of application is the recognition distance, meaning how far the user can be from the tag for the application to recognize it. A test was carried out to evaluate the performance of the application for the AR glasses Epson Moverio BT-200. Two A4 size images were placed on the wall (Figure 9). In the first experiment, the device was placed at 0.5 m from the image and was then moved away from the wall in increments of 0.5 m until the image was not recognized. In the second experiment, the device was placed at 5 m from the wall and then moved towards the wall in increments of 0.5 m. The detection distance depends on the device. The results can be seen in Table 4 and Table 5. In the worst case, it is necessary to be 1 m away from the target, but with an average camera, 1.5 m is ok.

The other devices, the mobile phones Huawei Honor 8, Samsung Galaxy s7, and Huawei P9, could recognize the images at a distance of up to 5 m.

We can conclude that, for bedbound patients, AR glasses Epson Moverio BT-200 are not suitable for use with the Vuforia library to recognize images in the walls because, normally, walls are more than 1 m away from the bed, and consequently, the glasses will not recognize the image. To recognize the image, the glasses should be no more than 0.5 m away from the image during the startup phase. Other devices such as the Huawei Honor 8, Samsung Galaxy s7, and Huawei P9 are suitable for this application (because they have higher-resolution cameras).

#### 6.2.7. Augmented Reality Test: Usability

This experiment focused on detecting if users prefer to use AR glasses or a mobile phone to control the room. A survey was carried out with 12 individuals from the health sector (five men and seven women), with ages from 22 to 54 years.

Two types of devices were used: augmented reality glasses (Moverio BT-200 from Epson) and a tablet device (Huawei Honor 8).

At the start of the test, each individual was provided with the user’ manual of the application printed on a paper. All participants were able to read it. Once the user read the manual, he or she was asked to wear the augmented reality glasses and to interact with the labels in the room. During the second phase, the user was asked to repeat the same actions while holding the mobile device. Once the second phase was completed, the subject was asked to fill in a questionnaire.

The results show that 92% of the users prefer the mobile phone over the AR glasses and that 100% think that the app will be useful for patients.

### 6.3. Discussion

The results show that the platform works well in a laboratory. The different communication protocols used (Z-Wave, 6LoWPAN, 802.15.4, Wi-Fi, and Bluetooth) are perfectly integrated thanks to the gateway and allow for an interconnection between devices and robots. All interfaces allow for room and robot control. Other architectures are based on a protocol, such as the ASTROMOBILE project [10], which uses Zigbee, or ENRICHME [43], which uses Z-wave. DOMHO and ECHONET use multiple protocols. Given the lack of dominant standards in smarthomes, we consider it important that architectures support multiple communication protocols.

As for multimodal interfaces, the results show that all of them can be used satisfactorily by healthy individuals. It is difficult to find in the literature a platform that simultaneously uses all five interfaces used in the architecture presented in this paper. Reference [32] presents eye, voice, and EMG gesture control of grasp with soft-robotic gloves. The results show that, on average, wink control required 1.2 ± 0.43 attempts to invoke opening or closing the glove. EMG gesture control required an average number of attempts of 1.4 ± 0.5. These are similar rates to the ones presented in our experiments. Reference [31] achieved 98% accuracy using special glasses with IR(Infrared).

Regarding gaze-based control, it has been found that there is no significance difference between wearing glasses and not wearing glasses. A minimum time for wink consideration was set to 0.22 s, and user preferences in terms of winking vs. staring were analyzed, concluding that most users prefer the staring mode. This differentiation is not made in the literature, with one or the other being used [32,44].

Regarding voice control, average times for performing actions (such as turning on a light) were established, which are related to the time it takes to navigate through the application menus. All actions are between 13 s and 41 s. It is also recommended to use pre-programmed commands (grammar files). No reference times are available in the literature.

We learned from gesture control that simple gestures are more recommendable as compound gestures are usually more difficult for older people and are more easily forgotten. In these, we obtain a reliability of 80%, better than the one reported by [32] of about 70%.

AR-based interfaces are interesting for people with mobility who can approach the photographs to interact with them, since sensing ranges are small in some devices. For people in a bed, they are not considered a good option. In general, users prefer cell phones to AR glasses.

## 7. Conclusions and Future Work

A new smart assistive environment architecture that integrates robotic systems and IoT devices was presented in this article. The architectures implements an ecosystem where smart devices using different communication protocols can interconnect. Service robots are also integrated into the architecture using an ROS interface. Therefore, the proposed architecture interconnects IoT environments, robots, and humans, creating a complete ambient assisted living environment. The interaction system was developed for a tablet PC held by an inherently safe assistive robotic arm. The design of this robotic arm, specifically designed for the application, is also presented in this article.

The architecture concept includes a complete and intuitive multimodal interaction system designed for smart rooms for people with certain disabilities or patients confined to a bed in their daily activities. This interaction system allows the user to control smart devices with different communication protocols in the room in five different ways: touch control, eye control, gesture control, voice control, and augmented reality control.

Different demonstrations of the use of the interaction system were studied and presented. The eye-based control shows that the user can control the room with eye clicking on the screen via staring or winking. Voice control and gesture control allow for controlling the room using upper limb gestures or voice commands. Finally, a new augmented reality interface for mobile devices or AR glasses was presented, with the users preferring the mobile device.

The architecture was implemented in a lab environment and tested with healthy subjects. Tests shows that the users consider the different interaction modes in a very positive way and that they can be adapted to people with different disabilities in the future.

Thanks to this architecture, users with various types of disabilities are able to interact with the devices in the room—automated bed, lights, shutters, climate, etc.—and with assistant robots, which are increasingly integrated in the medical environments, to ask for water, pills, newspapers, etc.

### Future Work

The work presented in this paper is the first step in the development of a smart assistive architecture for the integration of IoT devices, robotic systems, and multimodal interfaces in healthcare environments. The next steps that are under development are explained below.

At outset, tests will be conducted in controlled environments with people with disabilities. This will allow us to measure the usability of the interfaces with future users of the system. In a first phase, tests will be performed in the laboratory with handicapped people who can move independently. In a second phase, tests will be performed in medical environments or homes for patients with disabilities.

Secondly, we plan to address the issue of interoperability. Once devices from different networks are able to communicate with each other, it is necessary to follow high-level protocols such as IEEE 11073-20702 or the Personal Connected Health Alliance that allow interoperability with medical devices. It would be a first step towards the IoMT (Internet of Medical Things) [45]. It is also important to address the topics of edge computing and fog computing [46,47] in order to improve latency in communications and to reduce bandwidth.

Finally, we plan in the medium term to analyze and optimize the user experience, checking the usability by means of rigorous tools, such as self-report questionnaires, inventories, and symptoms checklists [48]. It is expected to improve the different user interfaces of the system (eye-tracking, gesture recognition, voice recognition, and augmented reality glasses) with newer devices and and to compare them to the state-of-the-art systems.

## Figures and Tables

**Table 1:** Accuracy and repeatability of the tests carried out with the robotic arm [3].

Test	Repetitions	Accuracy	Repeatability
1	10	<4 mm	<5 mm
2	9	<5 mm	<6 mm

**Table 2:** Users with glasses.

Tries/Subjects	1	2	3	4	5
1	166	151	299	123	106
2	199	118	522	122	135
3	181	91	600	78	136
4	131	92	417	121	105
5	135	77	375	77	104
6	147	77	282	120	124
7	198	137	284	122	121
8	152	91	408	168	120
9	151	105	550	106	105
10	136	136	409	80	104
Mean (ms)	159.6	107.5	414.6	111.7	116.0
STD (ms)	25.4	26.5	112.5	28.0	12.9

**Table 3:** Users without glasses.

Tries/Subjects	1	2	3	4	5
1	120	150	286	119	226
2	149	151	386	121	210
3	104	136	100	135	256
4	120	180	161	154	240
5	104	160	289	116	241
6	121	150	316	116	285
7	135	148	281	190	255
8	89	147	151	160	255
9	91	163	243	146	210
10	149	151	314	161	285
Mean (ms)	118.2	153.6	252.7	141.8	246.3
STD (ms)	20.5	11.2	84.2	23.5	25.2

**Table 4:** Results obtained moving towards the target starting at 5 m: NO means that the image was not recognized by the augmented reality (AR) device, and YES means that the image was recognized by the AR device.

Device/Distance (m)	5	4.5	4	3.5	3	2.5	2	1.5	1	0.5
Glasses AR Epson Moverio BT-200	NO	NO	NO	NO	NO	NO	NO	NO	YES	YES
Huawei HONOR 8	NO	NO	NO	NO	NO	NO	NO	YES	YES	YES
BQ E5	NO	NO	NO	NO	NO	NO	NO	YES	YES	YES
Samsung Galaxy S7	NO	NO	NO	NO	NO	NO	NO	YES	YES	YES
Huawei P9	NO	NO	NO	NO	NO	NO	NO	YES	YES	YES

**Table 5:** Results obtained moving away from the target starting at 0.5 m: NO means that the image was not recognized by the AR device, and YES means that the image was recognized by the AR device.

Device/Distance (m)	5	4.5	4	3.5	3	2.5	2	1.5	1	0.5
Glasses AR Epson Moverio BT-200	NO	NO	NO	YES	YES	YES	YES	YES	YES	YES
Huawei HONOR 8	YES	YES	YES	YES	YES	YES	YES	YES	YES	YES
BQ E5	NO	NO	YES	YES	YES	YES	YES	YES	YES	YES
Samsung Galaxy S7	YES	YES	YES	YES	YES	YES	YES	YES	YES	YES
Huawei P9	NO	YES	YES	YES	YES	YES	YES	YES	YES	YES

## Abbreviations

The following abbreviations are used in this manuscript:

6LoWPAN	IPv6 over Low-Power Wireless Personal Area Networks
AAL	Ambient Assisted Living
AR	Augmented Reality
EMG	Electromyography
GUI	Graphical User Interface
IoT	Internet of Things
IoMT	Internet of Medical Things
IP	Internet Protocol
IrDA	Infrared Data Association
LAN	Local Area Network
MQTT	Message Queuing Telemetry Transport
REST	Representational State Transfer
ROS	Robot Operating System
SDK	Software Development Kit)
TCP	Transmission Control Protocol
UAV	Unmanned Aerial Vehicle
VR	Virtual Reality
WPAN	Wireless Personal Area Networks
